# Visual motion does not bias gravity-referenced vestibular coding in the primate cerebellar nodulus and uvula

**DOI:** 10.1371/journal.pbio.3003972

**Published:** 2026-08-31

**Authors:** Lex J. Gómez, Robyn L. Mildren, Faisal Karmali, Kathleen E. Cullen

**Affiliations:** 1 Department of Biomedical Engineering, Johns Hopkins University, Baltimore, Maryland, United States of America; 2 Department of Otolaryngology-Head and Neck Surgery, Harvard Medical School, Boston, Massachusetts, United States of America; 3 Mass General Brigham, Massachusetts Eye and Ear, Boston, Massachusetts, United States of America; 4 Department of Otolaryngology, Johns Hopkins University, Baltimore, Maryland, United States of America; 5 Department of Neuroscience, Johns Hopkins University, Baltimore, Maryland, United States of America; 6 Kavli Neuroscience Discovery Institute, Johns Hopkins University, Baltimore, Maryland, United States of America; German Primate Centre Leibniz Institute for Primate Research: Deutsches Primatenzentrum GmbH - Leibniz-Institut fur Primatenforschung, GERMANY

## Abstract

Visual motion is known to influence perceptions of tilt, verticality, and translation, suggesting that optic flow is combined with vestibular cues to estimate orientation relative to gravity. The cerebellar nodulus and ventral uvula (NU) are a prime candidate to perform this computation because this region uniquely receives convergent semicircular canal, otolith, and proprioceptive inputs, and in non-primate species full-field visual motion robustly modulates NU activity. Here, we tested whether visual roll motion, known to bias perceived orientation relative to gravity, alters the internal gravity-referenced transformation used by NU neurons to encode vestibular self-motion. To test this, we recorded single-unit activity from NU Purkinje cells in rhesus macaques during whole-body translations in darkness, either without visual stimulation or after prolonged full-field optokinetic roll motion. We hypothesized that visual motion simulating head tilt would bias the NU’s internal gravity estimate, leading to altered translation-evoked responses. Contrary to this prediction, visual motion had no effect on either baseline firing rates or vestibular responses. Moreover, a computational model predicting visually induced shifts in neural tuning was not supported by the data. These results show that visual roll motion, although known to influence perceived orientation, does not bias gravity-referenced vestibular coding in the primate NU. This specialization may preserve a fast, body-anchored gravity estimate for postural and reflexive motor control, delegating visual–vestibular integration for perception to downstream circuits.

## Abstract 2

Visual motion can bias perceived orientation relative to gravity, raising the question of whether visual and vestibular signals are integrated within cerebellar circuits that encode self-motion. This study shows that visual roll motion does not alter Purkinje cell activity or gravity-referenced vestibular coding in the primate cerebellar nodulus and ventral uvula, indicating that visual influences on perceived orientation likely emerge in downstream circuits.

## Introduction

Maintaining a stable sense of gravity—and our orientation relative to it—is fundamental for every movement we make, from standing upright to navigating through the world. Human psychophysical studies show that visual motion, particularly global optic flow, can strongly bias perceived tilt, verticality, and translation, especially when vestibular cues are weak or ambiguous (human: [1–7]; non-human primate: [8]). These findings indicate that the brain combines visual and vestibular cues to construct gravity-referenced orientation estimates. Yet the neural circuits supporting this integration remain incompletely understood.

A central challenge for the brain is that visual motion is inherently ambiguous: it may result from self-motion or from movement in the external world—a problem known as motion-source separation [9]. In cortical circuits, this ambiguity is resolved by combining visual motion with self-motion signals—including vestibular and, when present, motor-related cues—such that congruent self-motion cues suppress vestibular responses, whereas incongruent cues enhance them [10]. Whether similar computations also occur in subcortical regions that are essential for spatial orientation is not yet known.

**Fig 1:**
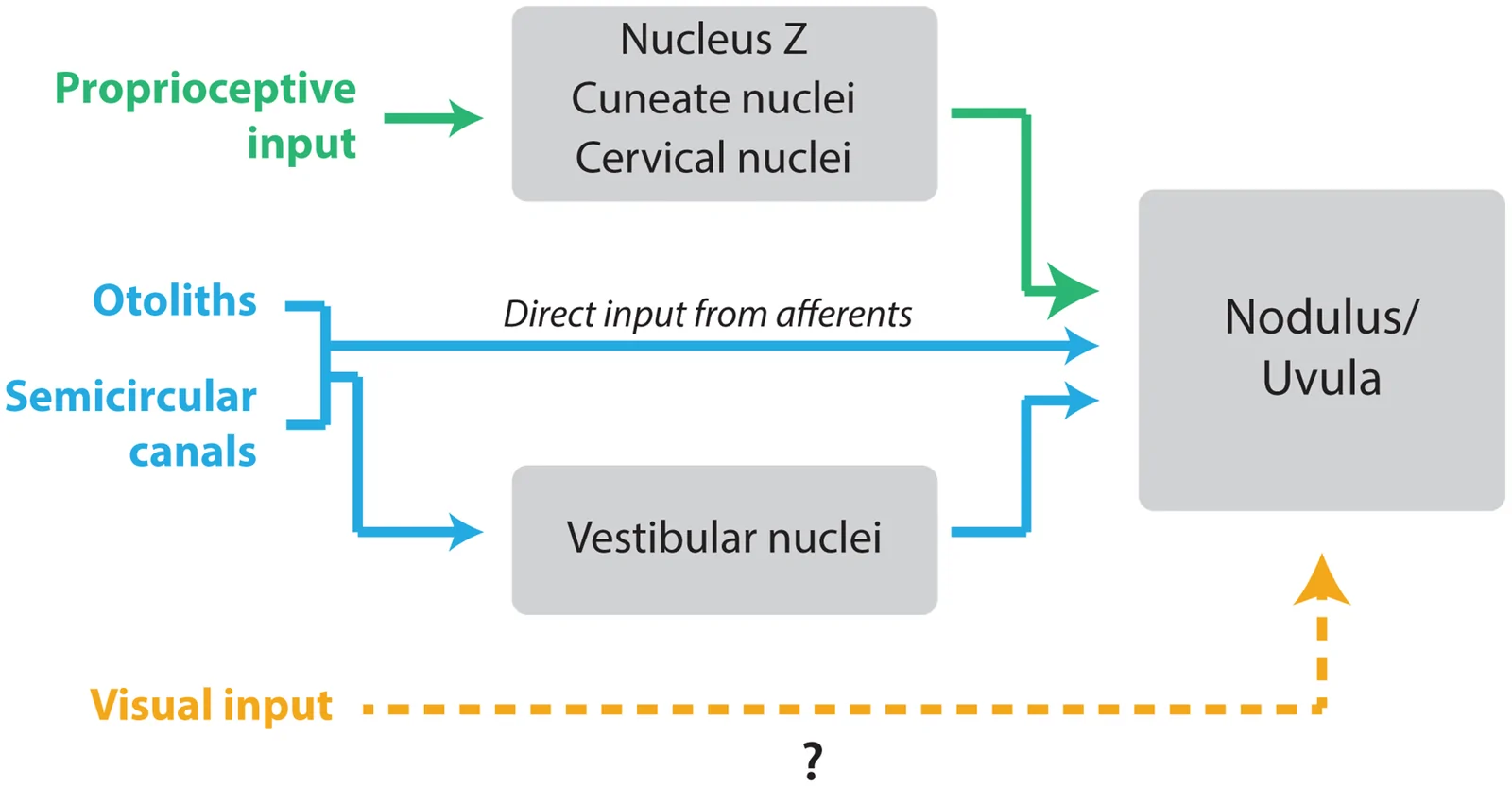
Estimating head orientation relative to gravity in the nodulus and uvula (NU). Diagram of known sensory inputs to the NU in non-human primate (blue and green arrows) and putative sensory input (dotted yellow arrow) that the NU may integrate to generate an estimate of head orientation relative to gravity.

Among these regions, the cerebellar nodulus and ventral uvula (NU; lobules X and IX of the posterior vermis) stand out as a likely candidate. The NU has long been recognized as a hub for estimating head orientation relative to gravity and for using this internal estimate to transform head-referenced vestibular signals into an earth-centered frame, thereby linking tilt and translation processing within a common gravity-referenced computation [11–13; reviewed in 14, 15]. It uniquely receives convergent mossy fiber input from semicircular canal and otolith afferents as well as proprioceptive signals from the neck and body [16–21]. Lesions to the NU abolish gravity-dependent reflexes, including postrotatory eye movements that normally realign gaze after changes in head orientation [13, 22], and disrupt whole-body stability and postural reflexes [23–26]. These anatomical and functional features suggest that the NU could integrate visual motion with vestibular and proprioceptive cues to construct a gravity-centered internal model of self-motion (Fig 1).

Neurophysiological evidence from non-primate species supports this possibility. In pigeons and rabbits, full-field visual motion robustly modulates NU Purkinje cell activity, and visual and vestibular inputs are integrated within a shared coordinate frame [27–30; reviewed in 31]. In primates, however, evidence is sparse and inconsistent: visual responses in cynomolgus macaques appear largely restricted to the dorsal uvula [32, 33], while in the rhesus macaque NU, Yakusheva and colleagues [34] reported only a small, midline-restricted population responsive to transient translational visual motion. Thus, whether visual–vestibular integration in the NU is a general feature across primates—or a species-specific specialization—remains unresolved (Fig 1).

Here, we directly tested whether visual motion cues modulate vestibular computations in the NU of rhesus macaques. Specifically, we tested whether visual roll motion consistent with head tilt biases the internal gravity estimate used by NU Purkinje cells, thereby altering their encoding of vestibular self-motion. We recorded Purkinje cell simple spike activity during whole-body translations in darkness, either without visual stimulation or after prolonged full-field optokinetic roll motion. In darkness, NU Purkinje cells displayed robust and heterogeneous responses to otolith stimulation, providing a rich neural basis for estimating head motion in space. Because visual roll motion strongly biases perceived tilt, we predicted that it would bias the NU’s internal gravity estimate and thereby alter the neural representation of the same physical translation within a gravity-referenced frame [11]. Contrary to this prediction, visual motion did not alter baseline activity or translation responses, indicating that the primate NU maintains a stable, visually independent estimate of orientation relative to gravity. This visual independence suggests that orientation estimates remain head- and body-centered within subcortical circuits, while visual integration likely occurs in higher-order cortical structures.

## Methods

### Experimental animals

All experimental protocols, housing, and surgical procedures were conducted in accordance with the National Institutes of Health (NIH) Guide for the Care and Use of Laboratory Animals and were approved by the Johns Hopkins University Animal Care and Use Committee (PR22M342). The cerebellar recordings were conducted in one male and one female adult rhesus macaque (*Macaca mulatta*). The monkeys were housed on a 12-hr light/dark cycle. Both monkeys had participated in previous studies in our laboratory and were in good health condition.

### Ethics statement

Consideration was given for the behavioral, emotional and social needs of the laboratory non-human primates when planning their housing such that compatible animals were housed in pairs and provided daily enrichment as per NIH guidelines. Throughout the study, animals were monitored in consultation with the Institution’s clinical veterinarian for any signs of change in their physiological and/or psychological state including: changes in responses to people, changes in levels of aggression, changes in quality and consistency of performance during experiments, changes in physical appearance (e.g., body weight and hair coat quality), loss of appetite, and the development of behavioral abnormalities. No such changes were observed. When endpoints were reached, animals were euthanized with a method that follows the best practices recommended by the NIH guidelines on: euthanasia of animals used in science is based on recommendations made by the International Council for Laboratory Animal Science (ICLAS) Working Group on Harmonization and the two international reference documents on euthanasia recommended by ICLAS: the American Veterinary Medical Association (AVMA) Guidelines on Euthanasia (2007) and the Working Party Report to the European Commission, Recommendations for euthanasia of experimental animals.

### Surgical procedures

The two monkeys were surgically prepared for extracellular recording using aseptic surgical techniques described previously [35, 36]. Animals were pre-anesthetized with ketamine hydrochloride (15 mg/kg i.m.); if necessary, Propofol (2–4 mg/kg, i.v.) was given to facilitate intubation. Buprenorphine (0.01 mg/kg i.m.) was administered to provide analgesia. Loading doses of dexamethasone (1 mg/kg i.m.) and cefazolin (50 mg/kg i.v.) were administered to minimize swelling and prevent infection, respectively. During surgery, anesthesia was maintained using 2%–3% isoflurane gas, combined with a minimum 3 L/min (dose adjusted to effect) of 100% oxygen. Heart rate, blood pressure, respiration, and body temperature were monitored throughout the procedure.

Surgery involved the implantation of a titanium headpost and stainless-steel or titanium recording chambers that allowed for targeting of the posterior cerebellar vermis. In the first animal, the positioning of recording chambers was determined using traditional stereotaxic targeting procedures; in the second animal, recording chambers were positioned based on the co-registration of CT and MRI scans using Brainsight (Brainsight 2 Vet, Rogue Research, Montreal, Canada). The implants were chronically fastened to the skull with titanium cortical screws (Veterinary Orthopedic Implants) and dental acrylic (OrthoJet, Wheeling, IL). A craniotomy was performed within the recording chamber placed over the cerebellum to allow electrode access to the cerebellar cortex. Following surgery, we continued dexamethasone (0.5 mg/kg i.m.; for 4 days), anafen (2 mg/kg day, 1 mg/kg on subsequent days), and buprenorphine (0.01 mg/kg i.m.; every 12 hour for 2–5 days, depending on the animal’s pain level). In addition, cefazolin (25 mg/kg) was injected twice daily for 10 days. Animals recovered for two (2) weeks before commencing experimentation. In both animals, a post-surgery CT scan was used to confirm accurate placement of recording chambers.

### Data acquisition

Kinematic data were collected via a 6D inertial measurement unit (IMU) and streamed to a data acquisition system (Blackrock Neurotech, Salt Lake City, UT) with a sampling rate of 1 kHz. We conducted acute recordings of extracellular neural activity in the NU using 128-channel read-write electrodes (IMEC, Columbia, MD). The 128 channels spanned a 1.6 mm recording area in a zig-zag pattern. Neural signals were amplified and bandpass filtered (0.1 Hz–7.5 kHz), digitized by four 32-channel RHS stim-recording head stages, and streamed at 30 kHz (Intan Technologies, Los Angeles, CA). A 0.1 Hz triangle wave signal was generated using a signal generator (Keysight Technologies, Columbia, MD) and recorded in both recording systems to enable later synchronization of data. For eye movement recordings, eye position was recorded using a high-speed camera (200 fps; Teledyne FLIR, Wilsonville, OR) and open-source software (Open Iris; [37].

### Experimental design

During the experiments, the monkey was head-fixed and seated in a primate chair secured to a custom-made linear sled platform, which allowed for whole-body translation in the mediolateral (ML) direction. The motion platform was centered within a 120 cm diameter recording chamber with a cylindrical wall. For visual stimulation, a spherical projector (TechnoConcept, Saint-Zotique, Québec) was used to project a rotating radial dot pattern on the cylindrical wall. The radial pattern covered 360°, with projected dots 3.8 cm in diameter and spaced 10 cm apart. We first assessed neuronal sensitivity to visual and vestibular stimulation when presented independently. To ensure recordings from unique populations of NU Purkinje cells across experimental sessions, electrodes were inserted acutely before each experiment using a custom microdrive (NaN Instruments, Nof Hagalil, Israel) and a sterile guide tube, and removed at the end of each session. During each recording, Purkinje cells were identified based on physiological signature and laminar position. As the electrode advanced from the molecular layer into the Purkinje cell layer, we observed prevalent complex spike activity in the dendritic region, followed by high-frequency simple spike activity at somatic depths, often still accompanied by identifiable complex spikes. We searched for and isolated neurons that showed modulation in response to mediolateral vestibular translations and recorded from responsive cells.

First, to test Purkinje cell responses to visual stimulation, we rotated the radial dot pattern along the roll, pitch, and yaw axes in turn, each at 60°/s. For all neurons tested in these axes (*N* = 32), we found they did not modulate their firing with visual rotation in the three axes examined. Next, we tested their baseline sensitivity to vestibular stimulation in the dark by applying whole-body translations on the linear sled in the mediolateral direction. The linear translations consisted of a transient displacement of 8 cm and peak velocity of 0.23 m/s, and 10 repeats were applied per direction (ipsilateral and contralateral relative to the recording side).

We then tested whether prior visual roll motion alters vestibular encoding in the NU. To accomplish this, we exposed monkeys to at least 50 s of visual stimulation about the roll axis, followed by vestibular stimulation (mediolateral translations). Roll visual motion was chosen because it can bias perceived orientation relative to gravity and therefore provides a targeted manipulation of the estimated gravity vector [1,2]. If visual cues update the NU’s internal gravity estimate, this manipulation should alter the gravity-referenced transformation of mediolateral translation signals. This visual stimulation again consisted of a rotating radial dot pattern projected on the wall of the recording chamber directly in front of the monkey. Ten repeats of translational stimulation were applied in each direction with a pause between translations (mean ~4.5 s). During the translations, the visual stimulation was briefly turned off (mean ~2 s) such that the vestibular stimulation occurred in the dark to prevent possible responses due to translation of the visual scene. This protocol was repeated for roll visual stimulation in both the clockwise (CW, −ive) and counterclockwise (CCW, +ive) directions at two different speeds (30°/s and 60°/s), with a rest period of 60 s in the dark after each condition. In a subset of neurons (13), the stimulus was only played at 60°/s, CW and CCW. For eye-movement recordings, this entire protocol was replicated but without applying mediolateral translations.

### Data analysis

#### Processing of kinematic and neural data.

All data were analyzed off-line. Kinematic data were imported into MATLAB (Mathworks, Natick, MA), and low-pass filtered using a Butterworth filter at 10 Hz. Neural data were first converted to bin files in MATLAB for spike-sorting. Neural data were spike-sorted in Kilosort version 2.5 [38], and then imported into the Phy2 python environment [39] for manual curation of putative spike waveforms. Waveforms of simple spikes were visualized in Phy2 and identified as single units, multiunit clusters, or noise. Briefly, units were classified as single units if they were well isolated across experimental conditions (i.e., exhibited minimal refractory period violations and stable firing rates across the recording session) and had typical waveforms; units were classified as multiunit clusters if they had typical waveforms but were not well isolated; and units were classified as noise if they met neither of these criteria. For all subsequent analyses, only single units were used (hereafter referred to as “units” or “unit activity”).

For eye-movement control sessions, the experimental protocol was replicated without mediolateral translations. To quantify persistence of the visual-motion response into the dark test intervals, we analyzed the magnitude of two-dimensional eye velocity, computed from the horizontal and vertical components, as a readout of optokinetic aftereffects following visual roll stimulation. Eye velocity was baseline-subtracted and normalized to the mean response during the immediately preceding the optokinetic stimulus (OKS) interval, such that baseline = 0 and the preceding OKS response = 1. Exponential fits to the post-stimulus decay were used to estimate OKAN time constants.

#### Vestibular- and visual-related neuronal discharge dynamics.

To characterize a unit’s vestibular sensitivity, we first computed continuous neuronal firing rates by applying a Kaiser filter with a cutoff window at 5 Hz to the spike trains, as previously described [40]. We then described each unit’s response to whole-body ML translation with a least-squares regression analysis:


$$\begin{array}{c} fr(t)=b+S_{v}\dot{X}(t)+S_{a}\ddot{X}(t)+S_{j}\dddot{X}(t) \end{array}$$ (1)

where *fr(t)* is the estimated firing rate at time *t*, b is the bias term, *S*_*v,*_ *S*_*a*_, and *S*_*j*_ are coefficients denoting the velocity, acceleration, and jerk sensitivities to whole-body translation at time *t*, respectively, and $\dot{X}$, $\ddot{X}$ and $\dddot{X}$, are velocity, acceleration, and jerk, respectively. For each coefficient in the analysis, we computed 95% confidence intervals using a non-parametric bootstrap approach (*n* = 2,000; [41, 42]). All non-significant coefficients were set to zero. We then used coefficients to estimate the sensitivity and phase of the response using the following equations:


$$\begin{array}{c} Sensitivity=sgn\left(S_{j},S_{a},S_{v}\right)\times \sqrt{\frac{(({\text{2}\pi f)}^{\text{2}}S_{j}-S_{v}{)}^{\text{2}}+(\text{2}\pi fS_{a}{)}^{\text{2}}}{({\text{2}\pi f)}^{\text{2}}}} \end{array}$$ (2)

$$\begin{array}{c} Phase=tan^{-\text{1}}\left(\frac{({\text{2}\pi f)}^{\text{2}}S_{j}-S_{v}}{\text{2}\pi fS_{a}}\right) \end{array}$$ (3)

where *f* = 1 Hz to match the duration of half-cycle of movements (500 ms) and the sign indicated whether the firing rate gain was positive (correlated with the stimulus) or negative (inversely correlated with the stimulus). Sensitivity is given in spikes per second per squared meters per second squared (written as (sp/s)/(m/s^2^)). The preferred direction of each cell (ipsilateral or contralateral to the recording site) was identified as the direction in which the neuron demonstrated the largest increase in firing rate. Preferred direction was determined based on sensitivity during the baseline condition (i.e., for translations in the dark).

To facilitate comparison with prior work [35], Purkinje cell responses to vestibular stimulation were categorized as linear, rectifying, or V-shaped based on their firing rate modulation relative to acceleration. Neural responses were first phase-aligned such that peak firing corresponded to the peak (or trough) of stimulus acceleration. Firing rate was then plotted as a function of acceleration to determine response symmetry and directional sensitivity. Cells were classified as linear if firing rates increased in the preferred direction and decreased in the non-preferred direction, with the absolute difference in directional sensitivities ≤8 (sp/s)/(m/s^2^). Cells were classified as rectifying if they exhibited an increase in firing rate in the preferred direction with a minimum sensitivity of 8 (sp/s)/(m/s^2^) and minimal modulation in the non-preferred direction (<8 (sp/s)/(m/s^2^)). Cells were classified as V-shaped if firing rates increased similarly in both directions (difference in sensitivities ≤8 (sp/s)/(m/s^2^)). Cells not meeting these criteria were categorized as other. The same classification procedure was repeated relative to velocity. In this case, identical symmetry criteria were applied, but the minimum modulation threshold was 30 (sp/s)/(m/s).

We next used two complementary approaches to determine whether NU Purkinje cells were modulated by constant-velocity visual roll motion. First, we applied the same dynamic linear regression model used to quantify vestibular sensitivity (Equations 1–2) to characterize each unit’s response to visual motion. In this context, *S*_*v,*_ *S*_*a*_, and *S*_*j*_ denote the velocity, acceleration, and jerk sensitivities to visual motion at time *t*, respectively, and $\dot{X}$, $\ddot{X}$ and $\dddot{X}$, represent the corresponding kinematic components of the visual stimulus. Visual sensitivity was expressed as (sp/s)/(m/s^2^). Because prior studies reported neural response latencies within the first 10 s of visual stimulus onset [27, 32, 33], regression analyses were performed using a window spanning 5 s before stimulus onset through the first 10 s of visual motion. Visual sensitivities were negligible; thus, preferred directions for visual motion could not be reliably determined, and units were grouped according to their vestibular preferred direction for analysis. Second, to assess whether visual motion altered baseline discharge, mean firing rates were computed over a 2 s window beginning 5 s prior to stimulus onset and at six subsequent time points following onset (0, 4, 8, 20, 40, and 50 s). We then tested for interactions between vestibular preferred direction and visual motion direction (clockwise, counterclockwise) and speed using linear mixed-effects modeling (see Statistical analyses).

To examine whether any modulatory effects of visual stimulation of vestibular responses were consistent across trials, we calculated each Purkinje cell’s sensitivity on a trial-by-trial basis (i.e., without bootstrapping). We then tested for interactions between vestibular preferred direction, visual motion direction (clockwise, counterclockwise), and speed using linear mixed-effects modeling (see Statistical analyses).

#### Modeling of dynamic changes.

To predict neuronal responses in the NU during translational motion when a rotational visual cue is provided, we developed a computational model. The key hypothesis driving this approach is that the rotational visual cue induces a tilt in the orientation estimate in the NU, and that this erroneous tilt would affect the transformation of lateral translation signals from head- to Earth-coordinates. Consequently, when the subject experiences a purely horizontal translation in Earth coordinates, the NU interprets the motion as a combination of horizontal translation and an artificial vertical component. Specifically, the stimulus to the otolith organs is purely horizontal in Earth coordinates. However, when the NU performs a transformation from head coordinates back to estimated Earth coordinates, this transformation incorporates the erroneously tilted reference frame, and thus, the NU estimate of motion has both a horizontal and a vertical component.

The specific effect of this transformation depends on the tuning direction of each NU neuron, and thus our modeling incorporates tuning direction (for further explanation, see Results). Under the assumption that visual rotation cues induce a roll shift in the coordinate frame about the *X* axis, the tuning directions of the neurons in the *Y* and *Z* directions are relevant. $\theta_{PD}\;$is the angle of the tuning direction in the *Y*–*Z* plane. Since rightward and upward translations are positive, 0° in the *Y*–*Z* plane points to the right, and CCW rotations are positive. For our modeled neurons (Fig 5), the sensitivity along the tuning direction in the *Y*–*Z* plane is $s_{PD}=\text{2}\text{0}(spikes/s)/(m/s^{\text{2}})$ and in the anti-preferred direction is $s_{APD}=\text{1}\text{0}\;(spikes/s)/(m/s^{\text{2}})$. The visual scene rotation induces an opposite-direction roll tilt. If this were to affect the coordinate transformation of linear acceleration signals, it is equivalent to a shift in the tuning direction in the opposite direction of the roll tilt, which is in the same direction as the visual scene rotation. This rotation of the tuning direction is assumed to be $\theta_{V}=\left\{-\text{2}\text{0}\circ ,-\text{1}\text{0}\circ ,+\text{1}\text{0}\circ ,+\text{2}\text{0}\circ \right\}$ for visual scene velocities of {−60°/s, −30°/s, +30°/s, +60°/s}, respectively. The adjusted tuning direction is $\theta_{PD}^{\prime }$=$\theta_{PD}$+$\theta_{V}$. The predicted responses in spikes per second (*fr*_*R*_) of the neurons to rightward stimuli are:

**Fig 2:**
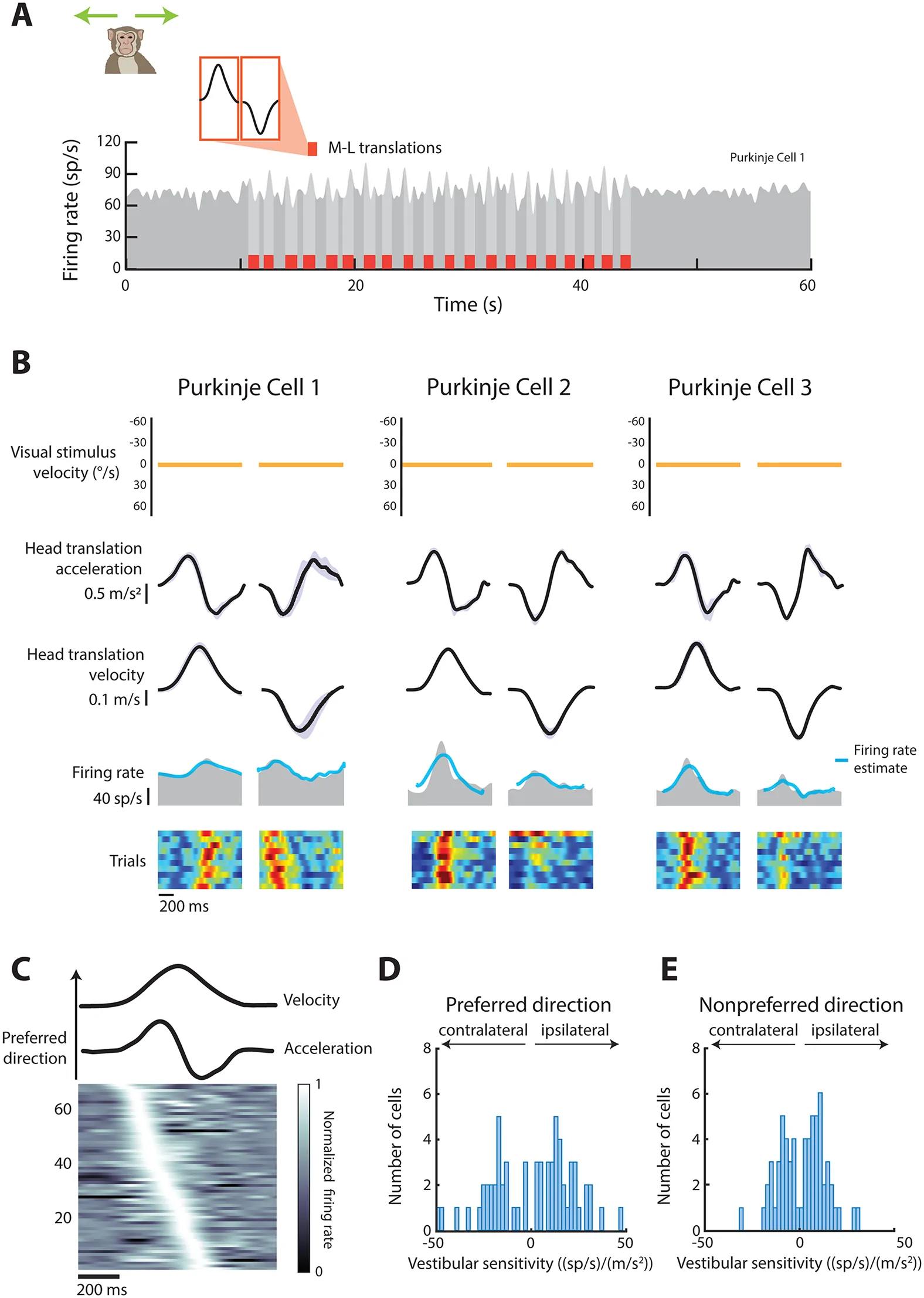
Vestibular responses of NU Purkinje cells in darkness. **(A)** Example Purkinje cell 1 firing rate during whole-body ML translation in the dark (gray shading); timepoints at which translations occurred are indicated by orange blocks. **(B)** Example tuning profiles for three Purkinje cells: linear (cell 1), rectifying (cell 2), and V-shaped (cell 3). Quantification across the population is shown in S1C and S1E Fig. For each cell, visual stimulus velocity (0°/s, darkness) is shown in the first row; head translation acceleration is shown in the second row; head translation velocity is shown in the third row; average firing rate (gray shading) along with the linear estimation of the firing rate based on head motion (superimposed blue trace) are shown in the fourth row; and heatmaps illustrating each example cell’s simple spike firing rate across trials are shown in the bottom row. **(C)** Heatmap showing the population response to vestibular stimulation in each neuron’s preferred direction. Phases tiled the cycle, ranging from acceleration-aligned to velocity-aligned dynamics. Quantification in S1A Fig. **(D)** Distribution of vestibular sensitivities to motion in the preferred direction. Positive and negative values represent cells with ipsilateral and contralateral preferences relative to the side of recording, respectively. **(E)** Same as in D, but for the non-preferred direction. https://doi.org/10.6084/m9.figshare.32836397.

**Fig 3:**
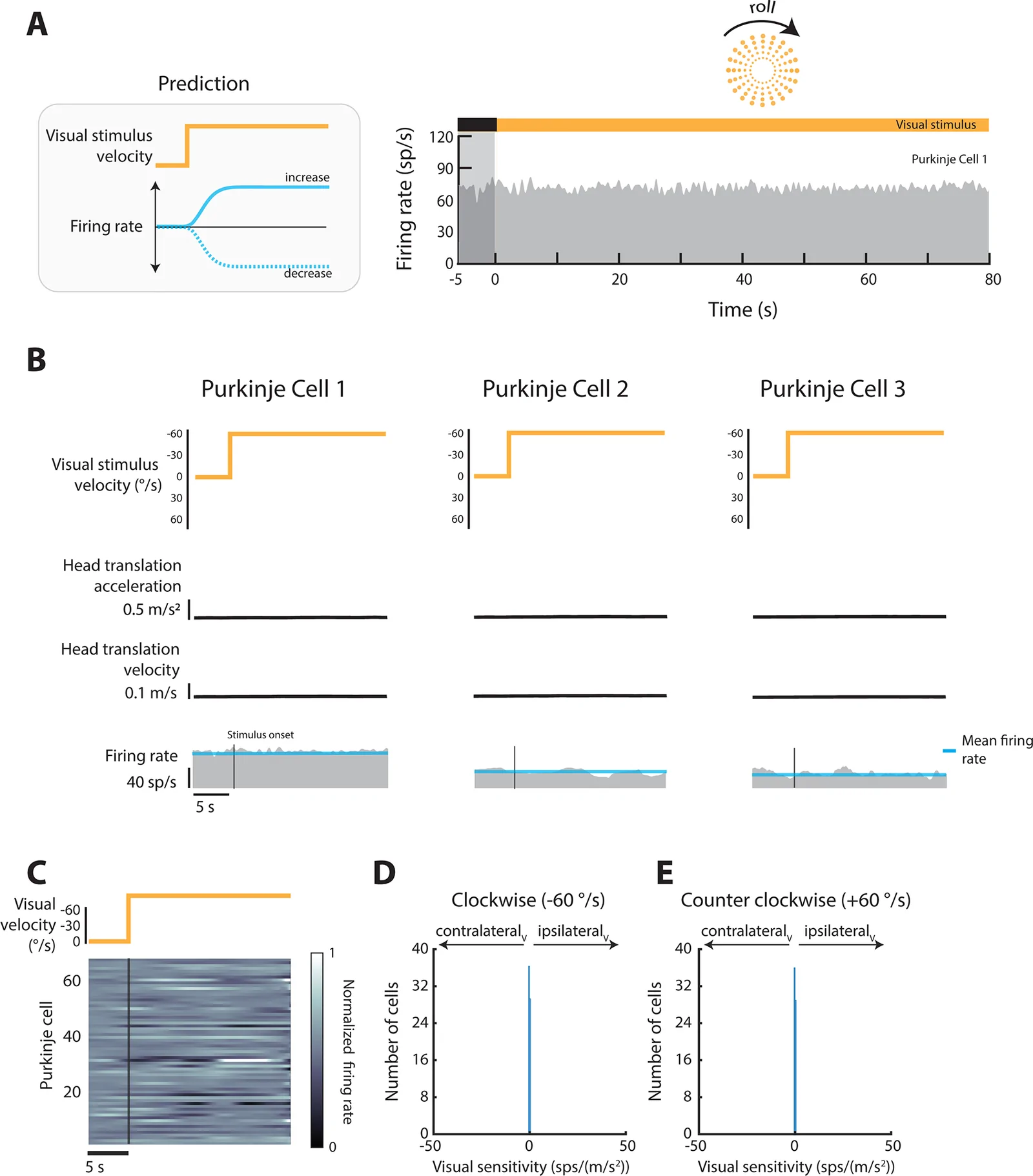
Visual roll motion does not modulate NU Purkinje cell baseline activity. **(A)** Left: Expected changes in firing rate at the onset of visual stimulus (based on findings from [32, 33]). Right: Example Purkinje cell 1 firing rate (gray shading) before and after the onset of full-field roll motion (−60°/s, clockwise). **(B)** Activity of the same three Purkinje cells as in Fig 2, before and after the onset of full-field roll motion (−60°/s, clockwise). Top row: visual stimulus velocity; second row: head translation acceleration; third row: head translation velocity; fourth row: average firing rate (gray shading) with mean firing rate (blue trace); bottom row: heatmaps of simple spike firing rates across trials. Each trial matched the duration of mediolateral translations in darkness, but in this condition the animal remained stationary while processing the visual stimulus. **(C)** Heatmap showing the population response to the trial-averaged normalized firing rate of each cell 5 seconds before stimulus onset and for the first 20 s of −60°/s visual stimulation, where cells are arranged based on the timing of their firing rate peak in the dark condition. **(D)** Distribution of sensitivities to clockwise visual stimulation (see Methods). Given the small magnitude of difference in the sensitivity between directions, these cells do not appear to fire with a true directional bias. Thus, to enable direction comparison with vestibular sensitivities, the signs of the visual sensitivities values were defined according to each cell’s preferred direction of vestibular stimulation. Positive and negative values represent preference for ipsilateral (ipsilateral_v_) and contralateral (contralateral_v_) vestibular stimulation, respectively. **E)** Same as in D, but for the counterclockwise stimulation; because the vestibular preferred direction remains the same, the distribution of signs does not change. https://doi.org/10.6084/m9.figshare.32836397.

**Fig 4:**
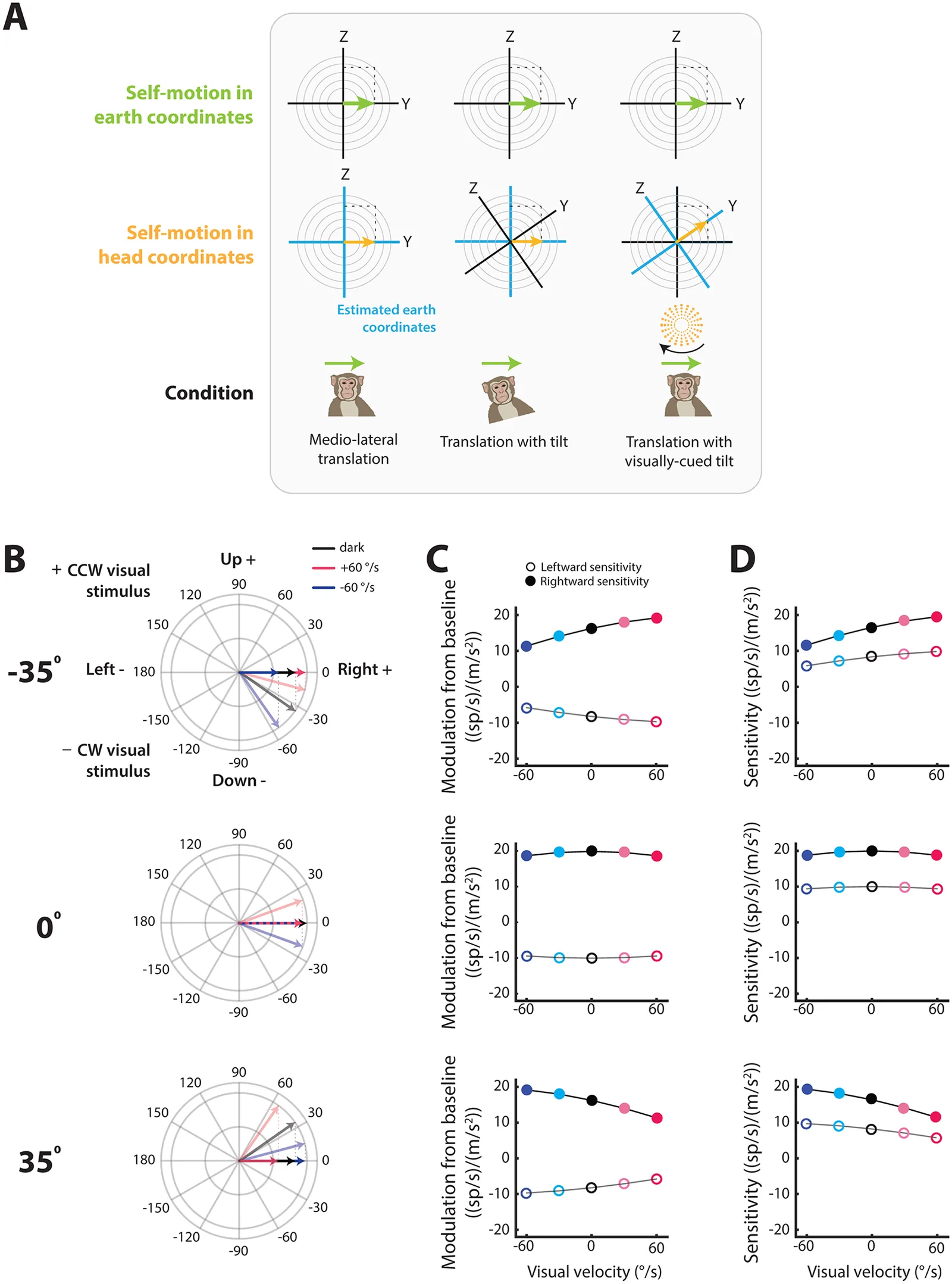
Model predictions of visual–vestibular interactions in NU Purkinje cells. **(A)** Schematic of self-motion in three coordinate conditions: Case 1—all coordinate systems aligned, self-motion vector identical across earth and head coordinates; Case 2—head tilted relative to earth, but the estimated earth coordinate system correctly aligned, yielding identical vectors; Case 3 (experimental condition)—visual motion erroneously rotates the estimated earth coordinate system, displacing the self-motion vector from real earth motion. Rightward and upward translations are positive, 0° in the *Y*–*Z* plane points right, and counterclockwise rotations are positive. **(B)** Model predictions of NU Purkinje responses in the frontal plane. Projection of the tuning direction in the *Y*–*Z* plane is shown as transparent arrows: dark gray = baseline tuning, pink = counterclockwise visual motion, light blue = clockwise visual motion. Opaque arrows show the projection onto the ML axis, indicating predicted responses to rightward motion with counterclockwise (red) and clockwise (dark blue) visual motion. Numbers (e.g., 35°) denote the polar angle of tuning direction; all example neurons shown are tuned for rightward motion. **(C)** Predicted response modulation relative to baseline firing as a function of visual scene velocity for each preferred direction angle, shown for rightward (filled circles) and leftward (open circles) motions. **(D)** Sensitivity, defined as response modulation adjusted for stimulation direction (sign reversed for leftward motions). https://doi.org/10.6084/m9.figshare.32836397.


$$\begin{array}{c} fr_{R}=\left\{ \begin{array}{c} s_{PD}\cdot cos\left(\theta_{PD}^{\prime }\right),\text{if the Y component of the tuning direction points rightward }(-\text{9}\text{0}\circ \leq \theta_{PD}^{\prime }\leq +\text{9}\text{0}\circ )\\ s_{APD}\cdot cos\left(\theta_{PD}^{\prime }+\text{1}\text{8}\text{0}\circ \right),\text{otherwise} \end{array}\right. \end{array}$$ (4)

The predicted responses in spikes per second (*fr*_*L*_) of the neurons to leftward stimuli are:


$$\begin{array}{c} fr_{L}=\left\{ \begin{array}{c} s_{APD}\cdot cos\left(\theta_{PD}^{\prime }+\text{1}\text{8}\text{0}\circ \right),\text{if the Y component of the tuning direction points leftward}(-\text{9}\text{0}\circ \leq \theta_{PD}^{\prime }\leq +\text{9}\text{0}\circ )\\ s_{PD}\cdot cos\left(\theta_{PD}^{\prime }\right),\text{otherwise} \end{array}.\right. \end{array}$$ (5)

Finally, the sensitivities in (sp/s)/(m/s^2^) to leftward (*S*_*L*_) and rightward (*S*_*R*_) stimuli are:


$$\begin{array}{c} s_{L}=\frac{fr_{L}}{\left(-\text{1}m/s^{\text{2}}\right)} \end{array}$$ (6)

$$\begin{array}{c} s_{R}=\frac{fr_{R}}{\left(+\text{1}m/s^{\text{2}}\right)} \end{array}$$ (7)

### Statistical analyses

All statistical analyses were performed in MATLAB. A Shapiro-Wilk test of normality was applied to all data; for analyses, non-normal data were log-transformed, and lognormality was confirmed using the Shapiro-Wilk test. For all statistical tests, *α* was set to 0.05; when appropriate, the Bonferroni correction procedure was used. Means are calculated from raw (non-normalized) data and reported with their standard error (SEM).

To test whether visual roll motion systematically altered Purkinje cell discharge rates over time as a function of vestibular preferred direction and the direction and speed of visual motion, we used linear mixed-effects modeling to account for repeated measurements within cells. Changes in firing rate were modeled with stimulus condition, preferred direction, and time as fixed effects, and random intercepts and slopes for time for each cell. Firing rates were log-transformed prior to analysis, and models were fit using restricted maximum likelihood. The significance of interaction terms was assessed using ANOVA on the fitted mixed-effects model. This same approach was used to test whether any modulatory effects of visual stimulation on vestibular responses were consistent across trials, applying the linear mixed effect model to vestibular sensitivities across movement trials. To determine if vestibular sensitivity was significantly altered by exposure to visual stimulation, we compared each cell’s sensitivity to vestibular stimulation in the dark with its sensitivity following +60°/s, +30°/s, −30°/s, and −60°/s visual stimulation using paired t-tests.

## Results

### Vestibular responses of NU Purkinje cells

To test the hypothesis that visual roll motion biases the gravity-referenced orientation estimate encoded by NU Purkinje cells, we first characterized their sensitivity to vestibular translation, determined whether visual roll motion alone altered baseline firing, and generated model-based predictions of how such a bias would manifest as changes in vestibular sensitivity. To establish a baseline for vestibular encoding in the primate NU, we recorded simple spike activity from 65 Purkinje cells during whole-body mediolateral (ML) translations in complete darkness (Monkey D: 30 cells; Monkey B: 35 cells). This condition isolates vestibular-driven responses by eliminating visual input, allowing us to assess how the NU represents linear head acceleration. An example response is shown in Fig 2A for a typical Purkinje cell, where firing rate modulations were consistently time-locked to translation onset across repeated trials.

Purkinje cells in our sample displayed heterogeneous tuning profiles to vestibular stimulation. Across the population, response phases spanned the stimulus cycle, ranging from dynamics most closely aligned with head acceleration to those aligned with head velocity, consistent with previous reports (Fig 2C; quantified in S1A Fig) [34, 35]. Consistent with prior descriptions of acceleration-based response categories [35], some cells exhibited linear responses across both movement directions, whereas others showed rectifying or V-shaped tuning (examples in S1B and S1C Fig). Importantly, comparable heterogeneity was observed when responses were phase-aligned to velocity (S1D–S1E Fig), indicating that this diversity is not specific to acceleration alignment. Together, this heterogeneity suggests that the NU population collectively encodes complementary aspects of translation dynamics, providing a distributed basis for estimating head motion in space.

We next quantified vestibular sensitivity by fitting a simple linear model relating the firing rate of each Purkinje cell to head movement kinematics [35,36]. For each cell, the “preferred direction” of translation was defined as the direction of motion that elicited the strongest increase in firing rate. We found that slightly more than half of our population (56.9%, 37/65 cells) preferred head motion in the direction ipsilateral to the side of recording, whereas 43.1% (28/65 cells) preferred contralaterally directed head motion. The distributions of sensitivities are shown in Fig 2D, where the mean sensitivity of cells with an ipsilateral preference was 16.8 ± 1.6 (sp/s)/(m/s^2^), and the mean sensitivity of cells with a contralateral preference was 21.1 ± 3.2 (sp/s)/(m/s^2^) (sensitivities in the non-preferred direction are shown in Fig 2E). These findings confirm that the primate NU implements a distributed population code for vestibular translation, spanning both kinematic and directional dimensions of self-motion.

### Visual motion does not alter baseline activity

To test whether visual roll motion biases the gravity-referenced orientation estimate encoded by NU Purkinje cells, we first asked whether visual motion alone modulates their baseline firing. Previous studies in primate and non-primate species have reported that NU Purkinje cells shift their resting discharge relative to baseline during sustained visual motion. Specifically, within the first 10 seconds of stimulus onset, firing rates can increase or decrease depending on stimulus direction and typically remain altered for the duration of stimulation [27,32,33]. Accordingly, if NU Purkinje cells were sensitive to visual roll motion, we expected sustained increases or decreases in baseline firing rate that depended on stimulus direction, as illustrated schematically in Fig 3A.

We exposed the monkeys to a radial dot pattern rotating clockwise (−CW) or counterclockwise (+CCW) about the roll axis at angular velocities of ±30°/s and ±60°/s (Fig 3A). A roll stimulus was chosen because visual stimulation in this axis can alter perceptions of orientation relative to gravity (i.e., cause a perceived tilt), including subjective visual vertical judgments in rhesus monkeys during roll optokinetic stimulation (human: [1–7]; non-human primate: [8]). Such visually induced biases in perceived orientation and optokinetic aftereffects are related manifestations of visual–vestibular processing and can persist following stimulus offset over time scales of several seconds [2,5,6,43]. To determine whether visual-motion processing persisted into the post-stimulus testing period, we quantified eye velocity during the brief dark intervals used for vestibular testing. Visual roll stimulation produced robust optokinetic aftereffects during these ~2-s intervals (S2 Fig), with measured proxy eye velocity remaining elevated relative to baseline rather than rapidly returning to baseline. In the −60°/s condition, exponential fits to the post-stimulus eye-velocity decay yielded time constants of approximately 4 s, exceeding the duration of the ~2-s dark intervals matched to vestibular testing. These aftereffects were present across repeated dark intervals, demonstrating that the visual-motion response induced by our stimulus persisted beyond stimulus offset and into the time window in which vestibular responses were measured.

Each stimulus was presented for ≥50 s while ensuring that the animal’s eyes remained open and directed toward the screen. For a subset of cells (*n* = 13), testing was restricted to the higher velocities (±60°/s). Fig 3B shows the activity of the same three example cells illustrated in Fig 2, now during the 5 s before −60°/s visual stimulus onset and for 20 s after onset. Although these Purkinje cells exhibited robust vestibular responses in the dark, none showed detectable modulation in response to visual motion. This lack of modulation was also apparent at the population level: Purkinje cell firing rates showed no stimulus-driven modulation (compare Fig 3C to vestibular responses in Fig 2C), and estimated sensitivity to visual motion was negligible (Fig 3D and 3E).

We quantified the absence of visual motion responses using two approaches. We first applied the same linear regression analysis used to characterize vestibular responses (see Methods). Unlike vestibular sensitivities, estimated visual motion sensitivities clustered tightly around zero (Fig 3D and 3E; −60°/s: 9.19 × 10^−^⁴ ± 9.10 × 10^−^⁵ (sp/s)/(m/s^2^); +60°/s: 6.95 × 10^−^⁴ ± 9.62 × 10^−^⁵ (sp/s)/(m/s^2^)). To then further assess whether stimulus direction or speed produced systematic changes in firing over time, we quantified each cell’s firing rate before stimulus onset, at onset, and at five subsequent time points during the 50-s priming period (S3 Fig). Cells were grouped by their vestibular preferred direction, and firing rates during visual stimulation were compared to pre-stimulus baseline using a linear mixed-effects model. We found no evidence of direction- or condition-dependent changes in firing rate over time (interaction terms: *F*_(3, 1622)_ ≤ 3.35, *p* ≥ 0.067). Thus, prolonged visual roll motion did not alter the baseline discharge of NU Purkinje cells.

### Model predictions of visual–vestibular interactions

The results of our experiments above showed that visual roll motion did not directly modulate NU Purkinje cell activity or baseline firing rate. Thus, we next asked whether such stimulation instead alters how these neurons encode vestibular inputs. Prior work has suggested that Purkinje cells use a central estimate of tilt relative to gravity to transform linear acceleration cues from a head-fixed to an Earth-fixed coordinate system (Fig 4A). Critically, when this tilt estimate is biased, for example by visual motion that produces an illusory sense of roll, the coordinate transformation itself should be rotated [44]. Under this framework, a virtual tilt should rotate the head-fixed coordinate system in the opposite direction of the perceived tilt; this would effectively change the projection of the tuning direction in the head-fixed coordinate plane, rotating it in the opposite direction of the virtual tilt. We therefore hypothesized that a visual roll stimulus generating erroneous tilt information would change the head-fixed coordinate frame of NU Purkinje cells, consequently modulating their responses to translation in the ML plane according to the direction (CW versus CCW) and intensity of the roll stimulus.

To quantify this hypothesis, we generated model predictions about the relationship between visual velocity cues, vestibular sensitivity to ML translations, and directional tuning in the *Y*–*Z* plane. We modeled cells that maximally increased their firing rate for translations in the *Y*–*Z* plane at $\theta_{PD}=\{$−70°, −35°, 0°, 35°and 70°} in darkness (i.e., in the absence of visual motion). Modeling results for −35°, 0°, 35° are plotted in Fig 4B (gray arrows). We then calculated how exposure to a visual roll stimulus would shift each model cell’s tuning direction in the *Y*–*Z* plane. For the purposes of modeling, we considered an idealized case in which neurons exhibited linear responses—firing rates increasing for translations along the tuning direction and decreasing in the opposite direction. While this simplification does not capture the diversity of response profiles observed across the population, it provides a useful framework to illustrate the predicted effects of visual roll motion on tuning direction. The results for the case where the tuning direction in darkness points directly right ($\theta_{PD}$=0°) illustrate the model’s predictions (Fig 4B, second row, black arrow). Because a visual roll stimulus should bias the tilt estimate in the opposite direction, CW visual motion would induce a CCW shift in perceived head tilt, effectively producing a CW rotation of the tuning direction (transparent blue arrows). Conversely, CCW visual motion would induce a CW shift in perceived head tilt, resulting in a CCW shift in tuning direction (transparent pink arrows). Notably, in the $\theta_{PD}$=0° case, the model predicts comparable firing rates for rightward translations during CW and CCW visual motion, with responses in both decreased relative to translations in the darkness. These modeling results thus emphasize that firing rate modulation due to ML translation and visual condition (CW, CCW, dark) should be interpreted in relation to each neuron’s underlying tuning direction.

Another illustrative case is a neuron with a $\theta_{PD}=$−35° (Fig 4B, first row, gray arrow). In this case the model predicts enhanced firing relative to baseline during rightward translation across all visual conditions. However, the degree of modulation depends on roll stimulus direction: responses are larger during +60°/s CCW visual motion (red arrow) and smaller during –60°/s CW visual motion (blue arrow), compared to the dark condition (black arrow). For the case $\theta_{PD}=$−70° the model makes similar predictions, with the important exception that for a visual velocity of −60°/s (CW), the adjusted tuning direction points directly downward, and thus the neuron is no longer predicted to respond to lateral motion (S4 Fig).

Fig 4C illustrates the model’s predicted response modulation for each neuron—defined as the change in firing rate from baseline, as a function of visual stimulus velocity for rightward (filled circles) and leftward (open circles) translations. In the dark, rightward translation evokes positive modulation (firing rate above baseline). This response becomes larger during CCW visual motion at +30°/s and +60°/s, and smaller during CW visual motion at –30°/s and –60°/s. In contrast, leftward translation produces negative modulation (firing rate below baseline) in the dark. Here, CW visual motion further decreases the response, whereas CCW visual motion increases it. Fig 4D shows the corresponding sensitivities for each neuron, calculated as response modulation normalized by stimulus direction (i.e., reversing the sign for leftward translations). Similar to response modulation, the predicted sensitivity patterns vary systematically with both visual velocity and tuning direction. Importantly, the trends for leftward and rightward translations mirror each other, underscoring that changes in vestibular sensitivity are tightly linked to the direction and magnitude of the visual roll stimulus.

### Visual roll motion does not alter vestibular sensitivity

**Fig 5:**
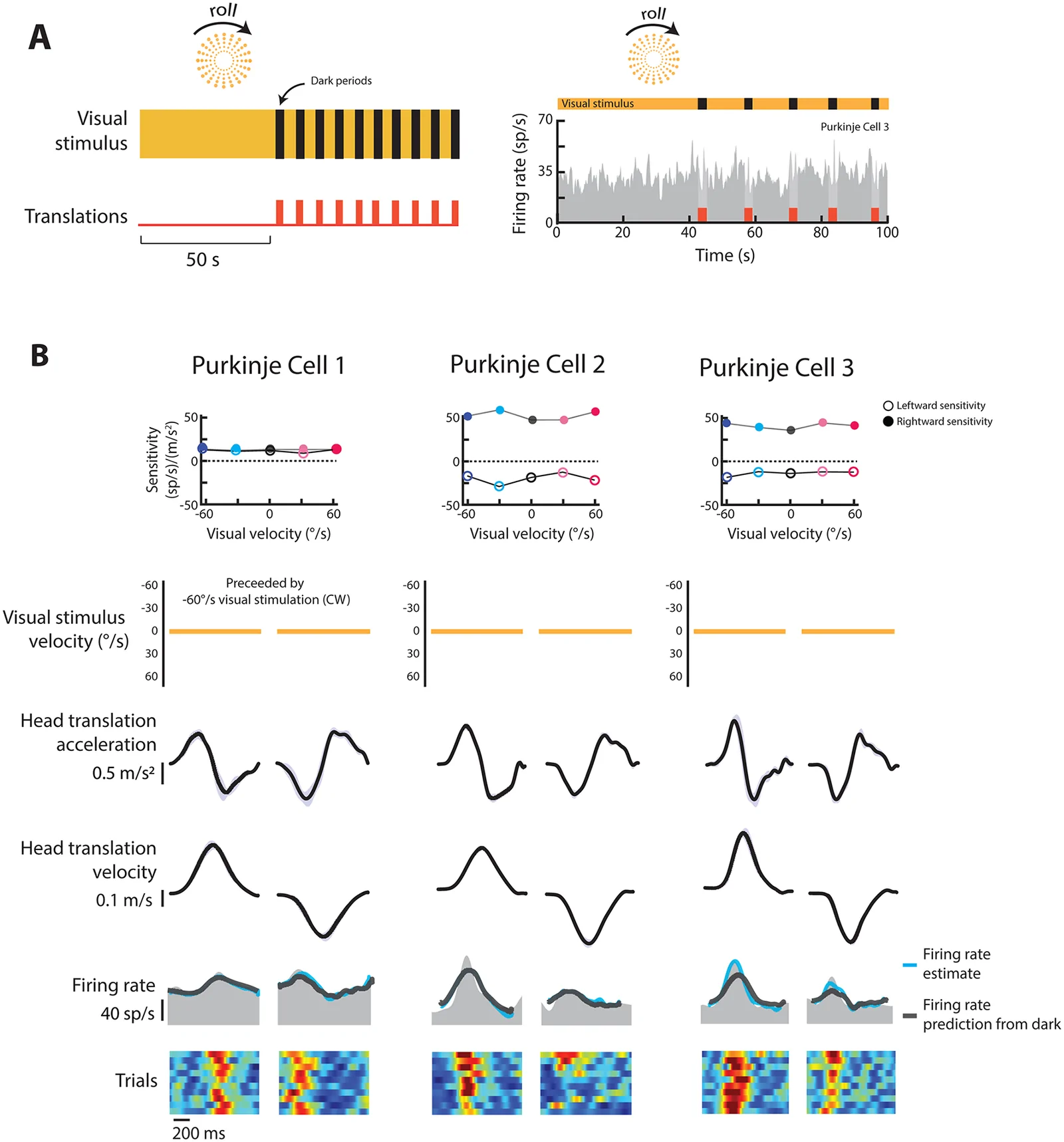
Testing model predictions: responses to vestibular stimulation following visual roll motion. **(A)** Experimental protocol: 50-s exposure to visual roll stimulus (±30°/s, ± 60°/s), followed by ML translations in darkness. Left: Example Purkinje cell firing rate (gray shading) following full-field roll motion (−60°/s, clockwise); timepoints at which translations occurred are indicated by orange blocks. **(B)** Average activity and heatmaps for the same three Purkinje cells as in Fig 2, following exposure to a visual roll stimulus (−60°/s, clockwise). Top row: sensitivity of the cell during rightward (filled circles) and leftward (open circles) translations following a visual stimulus rotating at −60°/s (dark blue), at −30°/s (light blue), translations in the dark, following a visual stimulus rotating at +30°/s (pink), and at +60°/s (red); second row: visual stimulus velocity at time of translations; third row: head translation acceleration; fourth row: head translation velocity; fifth row: average firing rate (gray shading) with linear estimate derived from head motion (blue trace); bottom row: heatmaps of simple spike firing rates across trials. Note: Error bars are omitted for sensitivities during each condition (top row). Because SEM is computed from 2,000 bootstrap resamples, the resulting variability is very low, and error bars would not be visible if plotted. https://doi.org/10.6084/m9.figshare.32836397.

To directly test these predictions, we next investigated whether visual roll motion biases vestibular encoding in NU Purkinje cells. We measured responses to ML translations immediately after a 50-s visual roll stimulus. The visual stimulus was briefly turned off during translation (Fig 5A), ensuring that any changes in neural activity would reflect shifts in the internal coordinate reference frame rather than direct visual influences. Fig 5B shows the activity of the same three example cells as in Figs 2B and 3B, now during vestibular stimulation after visual roll exposure. Sensitivity estimates for each condition are plotted above each cell (−60°/s, −30°/s, dark, 30°/s, 60°/s). None of these cells exhibited the distinct response patterns predicted by the model. For example, while the third cell displayed an upward trend for rightward (filled circles) motion with a clockwise visual stimulus, it did not have a corresponding trend for counterclockwise visual stimulation, or any trend for leftward motion (open circles). Across the population, we occasionally observed cells whose sensitivity in one translation direction appeared to vary with visual stimulus velocity or direction; however, these shifts were small, heterogeneous, and not systematic. Additional example cells are shown in S5 Fig. Together, these findings provide no evidence that visual rotational stimulation systematically alters translation responses in NU Purkinje cells.

Given that visual roll stimulation produced persistent oculomotor aftereffects throughout the dark intervals used for testing (S3 Fig), rapid decay of the visual-motion state is unlikely to explain the absence of visual modulation. To further test whether any effect was transiently present only during the earliest post-stimulus movements, we quantified neuronal sensitivity to head velocity on a movement-by-movement basis. Overall, sensitivity remained stable across movements (S6 Fig; interaction terms: *F*_(3,2154)_ ≤ 1.509, *p* ≥ 0.210), indicating that the absence of visual modulation was not explained by rapid decay of a post-stimulus effect.

Population-level comparisons of trial-averaged sensitivities further reinforced the lack of visual modulation of vestibular responses. Although we observed some variability in sensitivity across conditions (S7 Fig), there were no significant differences between responses to translations in the dark and those following visual stimulation in either the preferred (leftward-preferring cells: *t*_(27–35)_ ≤ 1.40, *p* ≥ 0.170; rightward-preferring cells: *t*_(23–28)_ ≤ 1.50, *p* ≥ 0.148) or the non-preferred direction (leftward-preferring cells: *t*_(27–35)_ ≤ 1.83, *p* ≥ 0.078; rightward-preferring cells: *t*_(23–28)_ ≤ 1.78, *p* ≥ 0.086, S8 Fig).

**Fig 6:**
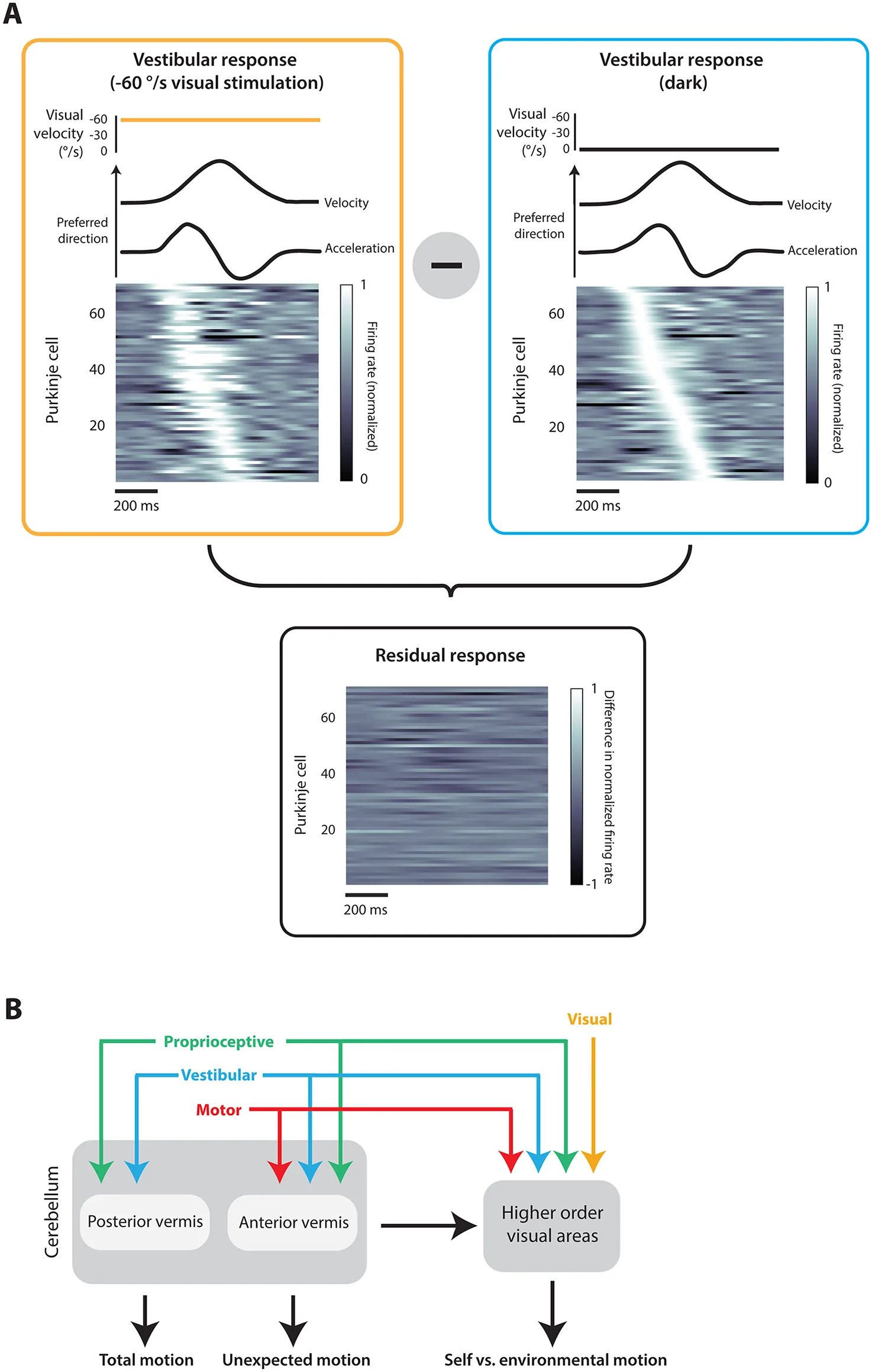
Invariant population coding across visual and dark conditions. **(A)** Left: Population heatmap of trial-averaged, normalized firing rates during vestibular stimulation following exposure to a −60°/s visual roll stimulus. Cells are ordered by the timing of their peak response in the dark condition; Right: Population heatmap of the same cells during vestibular stimulation in darkness (reproduced from Fig 2C); Below: Residual population activity obtained by subtracting responses to vestibular stimulation in the dark from vestibular stimulation following visual roll exposure, showing negligible differences between conditions. **(B)** Diagram of parallel streams of motion processing, showing sensory inputs to cerebellar structures and higher-order visual areas to compute different representations of motion.

Consistent with this, the firing rate responses of cells following −60°/s visual stimulation (Fig 6A) were nearly identical to those observed in the dark, with the residual activity showing minimal variation over the stimulation period. Whereas all cells (100%, 65/65 cells) exhibited responses exceeding threshold (see Methods) in the dark condition, the residual activity did so in only a negligible minority (6%, 4/65 cells) of cells during visual stimulation. Taken together, these results indicate that the vestibular sensitivity of NU Purkinje cells is not influenced by visual motion.

## Discussion

The central aim of this study was to determine whether visual motion signals bias vestibular computations in the primate nodulus and ventral uvula (NU)—a region of the cerebellar vermis that computes spatial orientation relative to gravity ([12,13,44]; also reviewed in [14, 15]). Human psychophysical studies have shown that full-field visual motion can strongly bias perceived orientation relative to gravity and influence self-motion perception [7], raising the possibility that visual cues might update the gravity-referenced internal model used by NU neurons to transform vestibular signals [11]. To test this possibility, we asked whether visual motion capable of inducing a perceived tilt alters the vestibular responses of NU Purkinje cells during translation, which provide a readout of the gravity-referenced transformation implemented by these neurons. Contrary to this prediction, neither baseline firing rates nor translation-evoked responses were altered following visual motion stimulation, and we did not observe any of the shifts predicted by our computational model. Together, these findings indicate that visual roll motion does not update the gravity-referenced transformation used by NU Purkinje cells. Thus, the primate NU appears to maintain a gravity estimate that is independent of visual motion, selectively integrating vestibular and proprioceptive cues.

### The case for visual–vestibular integration in the primate NU

Visual motion, particularly global optic flow, has long been known to alter perceived tilt, verticality, and translation, especially when vestibular cues are weak or ambiguous (humans: [1–7]; non-human primates: [8]). Such findings demonstrate that the brain normally integrates visual and vestibular signals to construct gravity-referenced perceptual estimates of self-motion.

At first glance, the NU seems an obvious substrate for such integration. The NU receives direct mossy fiber input from semicircular canal and otolith afferents [16, 21], proprioceptive input from the neck and body via the external cuneate nucleus and nucleus Z [17,20], and climbing fiber input from visually sensitive regions of the inferior olive [14,45–47]. Lesion studies underscore its functional importance, where damage to the NU abolishes gravity-dependent reflexes such as postrotatory eye movements [13,48,49] and destabilizes posture [23–26]. Moreover, neurophysiological evidence from non-primate species provides more compelling evidence of visual integration in the NU. Full-field visual motion robustly modulates nodulus Purkinje cell simple spikes in rabbits [27, 28] and complex spikes in both rabbits and pigeons are driven by climbing fiber inputs from visually responsive inferior olive regions [29,30,50–53], supporting integration of visual and vestibular signals in a shared gravity-referenced coordinate frame. In primates, evidence of visual integration in the NU has been more limited but still suggestive. Visual responses in cynomolgus macaques appear localized to the dorsal uvula [32, 33], and in rhesus macaques, Yakusheva and colleagues [34] reported a small midline-restricted population of NU cells responsive to transient translational visual motion.

Taken together, this convergence of psychophysical, anatomical, and physiological evidence provided a strong rationale for predicting that visual–vestibular integration would influence NU vestibular coding in primates. Yet, our recordings revealed no such effect. Constant-velocity visual motion, either alone or preceding translation, did not modulate the activity of vestibular-sensitive NU Purkinje cells. Across the population, neither baseline firing nor translation-evoked responses were altered, and the direction-dependent shifts predicted by our model were not observed (Figs 4B and S5). Thus, despite compelling reasons to expect visual influence, NU computations in rhesus macaques remain anchored to vestibular and proprioceptive input under conditions in which visual motion provides a strong orientation cue.

### Evolutionary divergence in NU visual processing

Placed in a comparative context, our results point to an evolutionary divergence in NU visual processing. In rabbits, full-field visual motion robustly modulates nodulus Purkinje cell simple spikes [27, 28], and complex spike responses in rabbits and pigeons are driven by visually responsive climbing fiber pathways [29,30,50–54]. These visual responses are directionally tuned to semicircular canal axes ([52]; reviewed in [31], indicating alignment in a shared coordinate frame for gravity-referenced orientation estimates.

Visual integration in mammalian NU, however, appears more variable. In cats, visual responses are minimal and show no vestibular interaction [55]. In primates, evidence is mixed: dorsal NU Purkinje cells in cynomolgus macaques respond to constant-velocity yaw-axis optic flow [32, 33], whereas in rhesus macaques (*Macaca mulatta*), Yakusheva and colleagues [34] identified only a small, midline-restricted population responsive to transient translational optic flow. Our present findings extend this view by showing no Purkinje cell responses to constant-velocity visual motion in any plane tested, and no effect of visual roll motion on subsequent vestibular sensitivity. Interestingly, a parallel species difference exists in the vestibular nuclei, where cynomolgus, but not rhesus, neurons integrate vestibular and proprioceptive cues despite receiving similar inputs [56]. Together, these findings suggest that NU visual integration varies across species, ranging from robust in birds and rabbits to minimal in rhesus macaques.

### Gravity estimation in the NU

Accurately estimating orientation relative to gravity is essential for balance and movement. No sensory organ directly encodes gravity; instead, the brain estimates gravity by integrating otolith inputs and angular velocity signals from the semicircular canals [57,58]. This allows the disambiguation of otolith signals, which reflect the net gravitational and inertial accelerations from both tilts and translations—two sources indistinguishable at the receptor level (Einstein’s equivalence principle; [59,60]). The NU is critical to this computation, with Purkinje cells tuned to tilt, translation, and composite gravitoinertial acceleration [11,34,49,61], supporting downstream representations in the fastigial and vestibular nuclei [12,62–64].

In addition to vestibular input, the NU receives proprioceptive signals from the neck and body [17,20] which are essential for transforming head-centered vestibular signals into a body-centered reference frame. Recent work further shows that NU vestibular responses are modulated by static head-on-body position in a gain-field-like manner [35] and that vestibular and proprioceptive inputs are summed synergistically, enabling downstream reconstruction of body-centered motion signals [44,49]. This integrative computation may be further shaped by upstream elements such as unipolar brush cells and molecular layer interneurons, which can temporally align canal and otolith signals ([16; reviewed in 15]). Accordingly, lesions of the NU not only disrupt both gravity-dependent eye movements but also profoundly impair the ability to maintain upright posture relative to gravity [13,22–26,65,66]. These deficits underscore the NU’s functional significance as a key cerebellar locus where vestibular and proprioceptive signals are combined to rapidly update stable representations of body motion and orientation, without relying on slower or more ambiguous streams of visual information.

### Functional dissociation between visual and non-visual gravity estimation

We propose that the absence of visual modulation in NU responses reflects a functional specialization for rapid, reflexive motor control. Unlike proprioceptive cues, visual motion is inherently ambiguous—it can result from either self-motion or movement in the external world. This “motion-source separation problem” [9] may make visual motion less suitable for directly updating fast vestibulo-ocular and vestibulo-spinal control signals. However, visual motion could still influence vestibular processing during simultaneous visual and vestibular stimulation, for example through gain control or divisive normalization mechanisms. Such mechanisms would reflect instantaneous multisensory interactions rather than a change in the underlying gravity estimate itself. By probing vestibular responses immediately following prolonged visual motion—after visual input was extinguished—we specifically tested whether visual cues altered the gravity-referenced transformation used to encode self-motion, independent of concurrent sensory interactions.

Importantly, visual roll stimulation produced persistent optokinetic aftereffects during the brief dark intervals matched to vestibular testing, indicating that visual-motion processing persisted beyond stimulus offset and into the relevant post-stimulus testing window. Although we did not directly measure perceived verticality during the neural recording experiments, prior psychophysical work in rhesus monkeys demonstrates that roll optokinetic stimulation can bias subjective visual vertical judgments, indicating that visual roll motion can influence gravity-referenced perceptual estimates in this species [8]. In addition, prior work in humans has shown that such visually induced biases in perceived verticality and tilt can persist after visual motion is interrupted [1,2], and that optokinetic aftereffects similarly persist in darkness following stimulus offset [43]. These findings establish that both perceptual and oculomotor consequences of visual motion can persist for several seconds after stimulus offset [2,5,6,43]. Consistent with this timescale, the optokinetic aftereffect in our eye-movement control data decayed with a time constant of approximately 4 s, exceeding the duration of the ~ 2-s dark intervals matched to vestibular testing. Moreover, vestibular sensitivity remained stable across successive post-stimulus movements, arguing against the possibility that an early visually induced effect was missed because it rapidly decayed. Together, these findings indicate that visual roll motion, despite producing persistent post-stimulus oculomotor effects, does not update the internal gravity reference frame represented in the primate NU.

This functional dissociation suggests that visual influences on perceived orientation are likely mediated outside the NU, in cortical or other parallel circuits. In primates, cortical regions such as the ventral intraparietal area (VIP; [67] and medial superior temporal area (MST; [68–74] integrate visual and vestibular signals in ways that are well suited for disambiguating self-motion. Related visuo-motor interactions have also been described in mouse primary visual cortex (V1; [10,75–77], although locomotion-related modulation of V1 activity appears substantially smaller in marmosets than in mice, indicating species-specific differences in cortical visuo-motor integration [78]. Thus, cortical circuits provide a plausible substrate for the flexible visual–vestibular integration required for perceptual inference under uncertainty.

Within the cerebellum, visual motion signals are also prominent, but they appear to support a different class of computations. In particular, Purkinje cells in the flocculus respond robustly to optokinetic stimulation across species [27,54,79,80], as well as to vestibular inputs ([81–84]; reviewed in [85]. These floccular responses are critical for adaptive gaze control and visually guided eye movements, but they serve a different function from the gravity-referenced transformation tested here. Consistent with this distinction, human studies show that visual cues can update perceived head orientation without influencing the orientation of rapid postural responses to vestibular perturbations [86]. Thus, visual motion clearly engages cerebellar circuits involved in gaze stabilization, but this does not imply that visual motion updates the NU-dependent estimate of body orientation relative to gravity.

At the level of cerebellar-brainstem motor pathways, sensory signals are rapidly compared with motor predictions to distinguish expected from unexpected motion, enabling fast reflexive corrections [87–92]. Our results suggest that visual motion does not update this rapid NU-dependent gravity estimate, likely because visual processing is slower and more ambiguous than vestibular and proprioceptive cues. Instead, convergence of vestibular and proprioceptive signals within the NU enables rapid computation of body motion and orientation for balance and reflexive control (reviewed in [35]. Together, these findings support a division of labor in which cortical circuits flexibly integrate visual motion for perception, floccular circuits use visual motion for gaze stabilization, and NU-dependent cerebellar–vestibular pathways prioritize fast, visually independent estimates of body motion and orientation for postural and reflexive control (Fig 6B).

## Supporting information

S1 Fig Response dynamics of NU Purkinje cells in response to mediolateral vestibular stimulation. **(A)** Stacked bar graph showing, for each neuron, the contribution of acceleration (blue), velocity (orange), and jerk (yellow) to the variance accounted for by the multiple linear regression model. **(B)** For each of the three example cells in Fig 2, the relationship between firing rate and the acceleration component of medio-lateral translations is shown. **(C)** Left: Scatter plot showing the acceleration-based sensitivities of each cell to ipsilateral motion compared to contralateral motion; cells are color-coded according to whether they met the criteria for being categorized as having a linear (purple), rectifying (blue), V-shaped (green) response. Right: Pie chart displaying the percent of neurons in each category in our sample population. **(D, E)** Same as in A and B, respectively, but now showing relationship of firing rate and sensitivity to velocity. (Note: In our population, jerk accounted for a minimal fraction of the variance accounted for relative to acceleration and velocity; thus, separate plots for jerk have not been included.) https://doi.org/10.6084/m9.figshare.32836397.
(EPS)

S2 Fig Visual roll stimulation produces persistent optokinetic aftereffects during dark intervals matched to vestibular testing. **(A)** Eye-movement control protocol for the 60°/s visual roll condition. Monkeys viewed full-field visual roll stimulation for ≥50 s, followed by repeated brief dark intervals (~2 s) matched to the timing of the neural vestibular test windows; no mediolateral translations were delivered. **(B)** Averaged two-dimensional eye-velocity magnitude pooled across clockwise (+60°/s) and counterclockwise (−60°/s) conditions, baseline-subtracted and normalized to the mean response during the immediately preceding OKS interval. Left: Eye velocity during the transition from preceding OKS interval to the first dark interval, aligned to visual stimulus offset. Middle: Same as the left panel, but for the transition to the second dark interval. Right: Same as left and middle panels, but mean eye velocity across test intervals 3–10. **(C)** Boxplots showing eye velocity during baseline, preceding OKS, and at each dark test interval. Values were baseline-subtracted and normalized to the mean response during the immediately preceding OKS interval, such that baseline = 0 and the preceding OKS response = 1. Outliers omitted for visualization. https://doi.org/10.6084/m9.figshare.32836397.
(EPS)

S3 Fig Visual roll stimulation has no effect on the resting discharge of NU Purkinje cells. **(A)** Illustration of firing rate over time (gray shading) from an example cell (Purkinje cell 1 from Figs 2 and 3) before and after the onset of +60°/s (CW) visual stimulation. Sample segments (timestamps) at different intervals are indicated by transparent magenta bars. **(B)** Boxplots displaying firing rates taken at each sampled time point after visual stimulus onset, normalized to the firing rate taken before stimulus onset for cells that preferred leftward translation during each visual stimulation condition (+60°/s: red; +30°/s: pink; −30°/s: light blue; −60°/s: dark blue). **(C)** The same as in B, but for cells that preferred rightward translation. https://doi.org/10.6084/m9.figshare.32836397.
(EPS)

S4 Fig Model predictions of visual–vestibular interactions in NU Purkinje cells with +/−70º tuning directions. **(A)** Model predictions of NU Purkinje responses in the frontal plane. Projection of the tuning direction in the *Y*–*Z* plane is shown as transparent arrows: dark gray = baseline tuning, pink = counterclockwise visual motion, light blue = clockwise visual motion. Opaque arrows show the projection onto the ML axis, indicating predicted responses to rightward motion with counterclockwise (red) and clockwise (dark blue) visual motion. Numbers (e.g., 70°) denote the polar angle of tuning direction; both example neurons shown are tuned for rightward motion. **(B)** Predicted response modulation relative to baseline firing as a function of visual scene velocity for each preferred direction angle, shown for rightward (filled circles) and leftward (open circles) motions. **(C)** Sensitivity, defined as response modulation adjusted for stimulation direction (sign reversed for leftward motions). https://doi.org/10.6084/m9.figshare.32836397.
(EPS)

S5 Fig Testing model predictions: responses to vestibular stimulation following visual roll motion for additional example cells. Left: Expected trends in sensitivity for leftward and rightward translations based on model predictions. Right: Sensitivity of eight example cells for both leftward (filled circles) and rightward (open circles) translations in each condition: following a visual stimulus rotating at −60°/s (dark blue), at −30°/s (light blue), translations in the dark, following a visual stimulus rotating at +30°/s (pink), and at +60°/s (red). https://doi.org/10.6084/m9.figshare.32836397.
(EPS)

S6 Fig Vestibular sensitivity remains stable across post-stimulus movement trials. **(A)** Normalized vestibular sensitivity of NU Purkinje cells with leftward translation preferences measured during each successive mediolateral translation following visual roll stimulation at +60°/s (red), +30°/s (pink), −30°/s (light blue), and −60°/s (dark blue). Sensitivities are normalized to each cell’s sensitivity during translation in darkness. Error bars indicate SEM. **(B)** Same as in A, but for cells with rightward translation preferences. **(C)** Normalized vestibular sensitivity pooled across leftward- and rightward-preferring cells and visual-stimulation conditions. Sensitivity remained stable across post-stimulus movement trials, including the earliest trials after visual-stimulus offset, arguing against the possibility that a transient visually induced modulation was missed because it rapidly decayed. Error bars indicate SEM. Outliers omitted for visualization. https://doi.org/10.6084/m9.figshare.32836397.
(EPS)

S7 Fig Visual stimulation does not systematically modulate NU Purkinje cell vestibular sensitivity. **(A–D)** Scatter plots depicting the distribution of sensitivities of NU Purkinje cells. Blue circles represent cells whose preferred direction is for leftward translation whereas red circles represent cells whose preferred direction is for rightward translation. For each cell, sensitivity to translations in the dark is plotted against sensitivity to translations following visual stimulation at +30°/s CW (A), −30°/s CCW (B), +60°/s CW (C), and −60°/s CCW (D). https://doi.org/10.6084/m9.figshare.32836397.
(EPS)

S8 Fig Visual stimulation does not systematically modulate NU Purkinje cell vestibular sensitivity in the non-preferred direction. **(A–D)** Scatter plots depicting the distribution of sensitivities of NU Purkinje cells in their non-preferred directions. Blue circles represent cells whose preferred direction is for leftward translation whereas red circles represent cells whose preferred direction is for rightward translation. For each cell, sensitivity to translations in the dark is plotted against sensitivity to translations following visual stimulation at +30°/s CW (A), −30°/s CCW (B), +60°/s CW (C), and −60°/s CCW (D). https://doi.org/10.6084/m9.figshare.32836397.
(EPS)

## Abbreviations

AVMA: American Veterinary Medical Association
CW: clockwise
CCW: counterclockwise
ICLAS: International Council for Laboratory Animal Science
IMU: inertial measurement unit
ML: mediolateral
MST: medial superior temporal area
NIH: National Institutes of Health
NU: nodulus and ventral uvula
SEM: standard error
VIP: ventral intraparietal area
